# IPP51, a chalcone acting as a microtubule inhibitor with *in vivo* antitumor activity against bladder carcinoma

**DOI:** 10.18632/oncotarget.4144

**Published:** 2015-05-15

**Authors:** Véronique Martel-Frachet, Michelle Keramidas, Alessandra Nurisso, Salvatore DeBonis, Claire Rome, Jean-Luc Coll, Ahcène Boumendjel, Dimitrios A. Skoufias, Xavier Ronot

**Affiliations:** ^1^ Université Joseph Fourier, AGIM CNRS FRE, EPHE, GRENOBLE Cedex 9. Université Joseph Fourier, Grenoble, France; ^2^ Unité INSERM/UJF U823, Centre de recherche Albert Bonniot, Grenoble, France; ^3^ School of Pharmaceutical Sciences, University of Geneva, University of Lausanne, Quai Ernest-Ansermet, Geneva, Switzerland; ^4^ UMR, Institut de Biologie Structurale J.-P. Ebel, Grenoble, France; ^5^ Unité Inserm, Grenoble Institute of Neuroscience, Site Santé, Grenoble, France; ^6^ Université de Grenoble/CNRS, UMR, Département de Pharmacochimie Moléculaire, Grenoble Cedex, France

**Keywords:** microtubule inhibitor, bladder cancer, antitumor agent, flavonoid, mitosis

## Abstract

We previously identified 1-(2,4-dimethoxyphenyl)-3-(1-methylindolyl) propenone (IPP51), a new chalcone derivative that is capable of inducing prometaphase arrest and subsequent apoptosis of bladder cancer cells. Here, we demonstrate that IPP51 selectively inhibits proliferation of tumor-derived cells versus normal non-tumor cells. IPP51 interfered with spindle formation and mitotic chromosome alignment. Accumulation of cyclin B1 and mitotic checkpoint proteins Bub1 and BubR1 on chromosomes in IPP51 treated cells indicated the activation of spindle-assembly checkpoint, which is consistent with the mitotic arrest. The antimitotic actions of other chalcones are often associated with microtubule disruption. Indeed, IPP51 inhibited tubulin polymerization in an *in vitro* assay with purified tubulin. In cells, IPP51 induced an increase in soluble tubulin. Furthermore, IPP51 inhibited *in vitro* capillary-like tube formation by endothelial cells, indicating that it has anti-angiogenic activity. Molecular docking showed that the indol group of IPP51 can be accommodated in the colchicine binding site of tubulin. This characteristic was confirmed by an *in vitro* competition assay demonstrating that IPP51 can compete for colchicine binding to soluble tubulin. Finally, in a human bladder xenograft mouse model, IPP51 inhibited tumor growth without signs of toxicity. Altogether, these findings suggest that IPP51 is an attractive new microtubule-targeting agent with potential chemotherapeutic value.

## INTRODUCTION

Microtubules, composed of αβ-tubulin heterodimers, are key components of the cytoskeleton. Microtubules form hollow rods and are essential in a variety of fundamental processes, such as cell division, intracellular transport, maintenance of cell shape and polarity [1]. Microtubules constantly undergo phases of polymerization and depolymerization, and these dynamics are important for many cellular processes, the most dramatic of which being mitosis. Several natural and synthetic drugs inhibit microtubule dynamics leading to mitotic arrest and subsequent cell apoptosis. For example, paclitaxel, vinca-alkaloids and epothilones are currently used to treat a variety of human cancers [2]. However, their clinical success is often limited by severe side effects, such as neurotoxicity, poor accessibility of the drug due to multidrug resistance transporters and drug resistance due to tubulin mutations [2, 3]. Therefore, there is a continuing need for the development of novel anti-microtubule drugs.

Flavonoids are naturally occurring polyphenols that are widely distributed throughout the plant kingdom. They are classified into several subclasses, including chalcones, flavones, flavanones, flavonols and aurones. In plants, they are biosynthesized through a key step involving the cyclization of chalcones (1,3-diphenylpropanoids), which are flavonoid precursors. Flavonoids are frequently recommended as preventive agents against several diseases. For instance, Asians, who ingest more flavonoids in their diet than populations in the Western hemisphere, have much lower risks of colon, prostate and breast cancers, and this raises the question of whether flavonoids can serve as natural chemopreventive and anticancer agents [4-6]. Various molecules derived from flavonoids have been studied *in vitro* and *in vivo* to determine their potential anticancer activities [7, 8]. For example, quercetin, a natural flavonol, is an inhibitor of different kinases involved in cancer progression, such as epidermal growth factor receptor (EGFR), cyclin-dependent kinases and Aurora-A [9, 10]. Clinical trials were performed on quercetin and have yielded encouraging results, but further studies are needed to determine its possible use in adjuvant cancer therapy [11, 12]. Flavopiridol, a semi-synthetic flavone, was the first inhibitor of cyclin-dependent kinases tested in human clinical trials to treat chronic lymphocytic leukemia patients [13, 14]. Clinical phase I/II trials have demonstrated the efficacy of flavopiridol in high-risk chronic lymphocytic leukemia patients, although side effects, such as hyperacute tumor lysis syndrome, limit its use [15]. However, the administration of flavopiridol following a new schedule was recently shown to be effective and to have limited toxicity for the treatment of non-Hodgkin's lymphoma [16]. While these studies demonstrated that tested flavonoids retain their anticancer properties *in vivo*, they also showed that the bioavailability of flavonoids are often limited and that efficacious doses often lead to unwanted side effects, such as liver failure and anemia [17]. In this context, our goal was to develop more efficient flavonoids that are active against bladder carcinoma [18-21]. Bladder cancer is the 10th most common cancer worldwide, with the highest rates being reported in Europe, North America and Australia, and it caused approximately 150,000 deaths worldwide in 2008 [22]. Age is a notable risk factor for the development of bladder cancer, but the greatest risk is exposure to aromatic amines, which are found in the products of chemical industries and in hair dyes, paints and cigarette smoke [23]. Approximately 70% of newly diagnosed bladder tumors are non-muscle-invasive, but approximately 50-70% of these tumors recur and 10-20% progress to muscle-invasive disease [24]. Since the 1980s, the standard treatment for advanced bladder cancer has been MVAC, a combination of methotrexate, vinblastine, doxorubicin and cisplatin [25]. This regimen results in a median survival duration of 13 to 15 months, and no other drug combination has generated a better survival rate [26]. Despite its clinical activity, the MVAC regimen has some limitations, such as a relatively short response duration and significant toxicities. Bladder cancer poses a significant economic burden for the following reasons: the need for lifetime surveillance, the treatment of recurrent tumors, and the cost of complications associated with treatment; thus, new chemotherapeutic agents are urgently needed [27].

**Figure 1:**
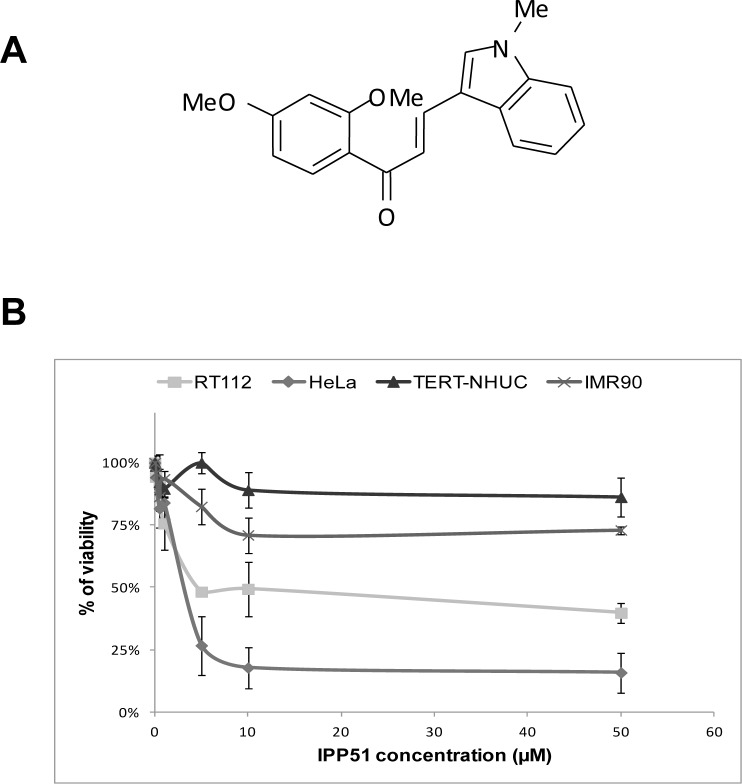
IPP51 selectively inhibits proliferation of human cancer cells versus normal non tumoral cells **A.** Chemical structure of IPP51. **B.** The cell proliferation MTT assay was done after 48 h of treatment with various concentration of IPP51. The results were expressed as a percentage of the vehicle treated cells. Data shown are the mean ± SD of three independent experiments.

We have recently identified 1-(2,4-dimethoxyphenyl)-3-(1-methylindolyl) propenone (IPP51) (Figure 1A), a new chalcone derivative that is able to inhibit bladder cancer cell proliferation [19]. Chalcones target different cellular proteins, such as tubulin, phospholipase A2 and DNA topoisomerase [28-30]. IPP51 is a member of the methoxylated chalcones, which are known to be potent antimitotic agents [31, 32]. Interestingly, it is known that inhibitors of tubulin polymerization, such as combretastatin A and colchicine, are also rich in methoxy groups [33, 34]. The antimitotic activity of chalcones could also be attributed to the enone moiety of these molecules, which can interact with a critical thiol residue in the colchicine binding site of tubulin [35, 36]. Indeed, we have shown that IPP51 treatment induces cell cycle arrest at the G2+M phases, which leads to apoptotic cell death through caspase-3 activation. The antimitotic effect of IPP51 on bladder cancer cell lines, the fact that indolyl-appended chalcones have never been investigated as colchicine-like agents and the ease with which IPP51 was synthesized prompted us to further characterize the mechanism of action of IPP51 and its potential interaction with tubulin. In the present study, we show that IPP51 binds to the colchicine binding site of tubulin and inhibits the polymerization of microtubules both *in vitro* and *in cellulo*. Furthermore, the results of our *in vivo* studies in mice show that IPP51 can inhibit the growth of bladder cancer xenografts. Overall, our results demonstrate that IPP51 is a novel anti-microtubule agent.

## RESULTS

### Selective effect of IPP51 on tumor cells

Our chalcone-derivative IPP51 was previously tested in different bladder cancer cell lines isolated from bladder tumors of different grades and stages that mimic the bladder tumor progression process [19]. The IC_50_ of IPP51 (concentration resulting in 50% loss of cell viability) was found to be 5 μM for cell lines derived from low grade tumors (RT4 and RT112 cell lines) and approximately 50 μM for cell lines derived from high grade tumors (T24, TCCSUP and J82 cell lines). To determine if IPP51 had specific activity against tumor cells, it was tested in HeLa cells (derived from a cervical cancer) and immortalized normal human cell lines: urothelial cells, the TERT-NHU cell line [44] and human fetal lung fibroblasts, the IMR-90 cell line. As observed previously in bladder cancer cells, IPP51 inhibited HeLa cell proliferation in a dose-dependent manner, with an IC_50_ value of 4.4 ± 0.4 μM. Interestingly, IPP51 did not affect the proliferation of TERT-NHU cells at 5 μM, which is its IC_50_ for RT112 cells (Figure 1B). Importantly, even at the higher IPP51 concentration (50 μM), it remained inactive against TERT-NHU cells. Furthermore, IPP51 had little effect on IMR-90 cell proliferation. Even at IPP51 concentrations up to 50 μM, the viability of IMR-90 cells was approximately 75%. This observation encouraged us to further analyze the mechanism of action of IPP51 because it appeared to be a potent selective inhibitor of tumor-derived cell lines.

### IPP51 disorganizes mitotic spindles

**Figure 2:**
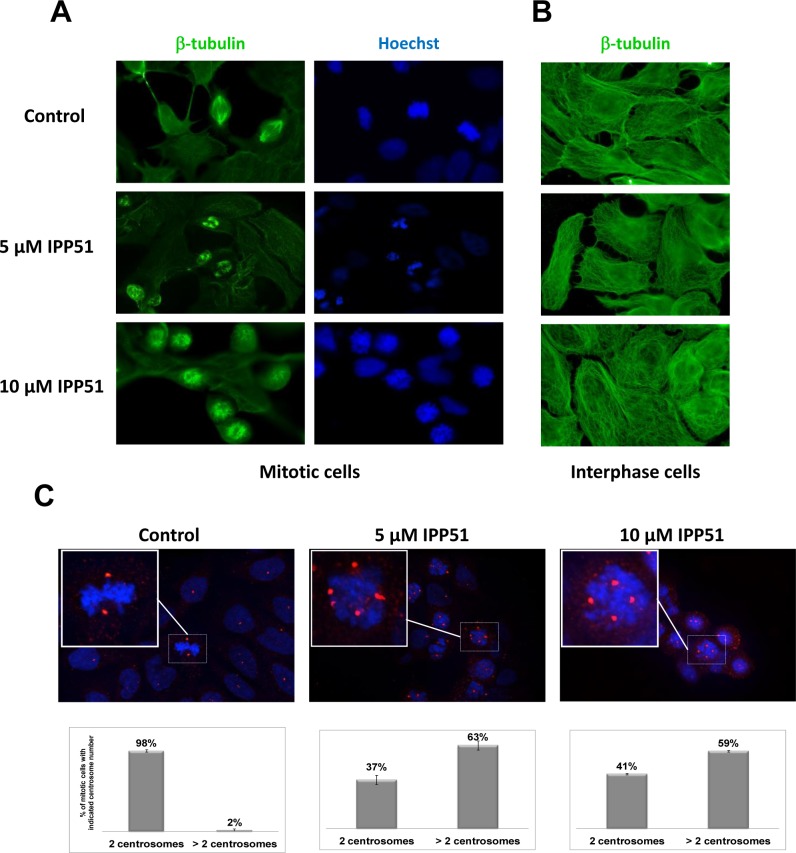
IPP51 disrupts mitotic spindle formation **A.** and **B.** RT112 cells treated with indicated concentrations of IPP51 for 16 hours were stained for β-tubulin (green) (left and middle columns) and Hoechst (blue) (right column). Micrographs show representative interphase **A.** or mitotic **B.** cells. **C.** *Upper*: Representative immunostaining for γ-tubulin (red spots) of RT112 cells incubated with vehicle (Control) or IPP51 for 16 hours. Insets show a zoom of boxed area. *Bottom*: Number of γ-tubulin spots was counted on at least 100 mitotic cells from different microscope fields randomly selected. The data represented are the mean ± SD of three independent experiments.

IPP51 was previously shown to induce an increase in the proportion of RT112 cells arrested at the G2+M phase of the cell cycle [19]. The same effect was observed in HeLa cells (data not shown). Interference with mitotic progression is often associated with perturbation of microtubule dynamics. Therefore, we tested whether IPP51 affects microtubule organization in dividing cells by examining HeLa cells stably expressing GFP-tubulin by videomicroscopy. Following the addition of 10 μM IPP51, preformed bipolar spindles began to dismantle, spindle microtubules shortened and the spindle poles split into multiple asters. Compared to control cells, IPP51-treated cells showed clear spindle assembly defects and failed to proceed into anaphase and cytokinesis (Supplementary Movies S1 and S2). Similar aberrant spindles were observed in IPP51-treated RT112 cells by immunofluorescence microscopy (Figure 2A). In the presence of IPP51, all mitotic spindles were aberrant, as indicated by multiple short microtubule asters, misaligned chromosomes and defects in DNA congression to the metaphase plate. In contrast, untreated cells had normal bipolar spindles with chromosomes aligned to the metaphase plate (Figure 2A). Interestingly, at the IPP51 concentration that induced aberrant spindles, the interphase microtubule network appeared to be unaffected compared to that of untreated cells (Figure 2B). Centrosomes, which are duplicated before mitosis, control spindle organization and ensure spindle bipolarity during mitosis [45]. Among their components, γ-tubulin is the most well-characterized and is known to promote microtubule nucleation from the centrosomes [46]. As expected, two spots of γ-tubulin were visualized in 95% of mitotic cells in the control, leading to the formation of normal bipolar spindles (Figure 2C). However, in presence of IPP51, approximately 60% of mitotic cells showed supernumerary microtubule organizing centers with three or more γ-tubulin spots, leading to multiple spindle asters (Figure 2C).

### IPP51 treatment induces mitotic checkpoint activation

**Figure 3:**
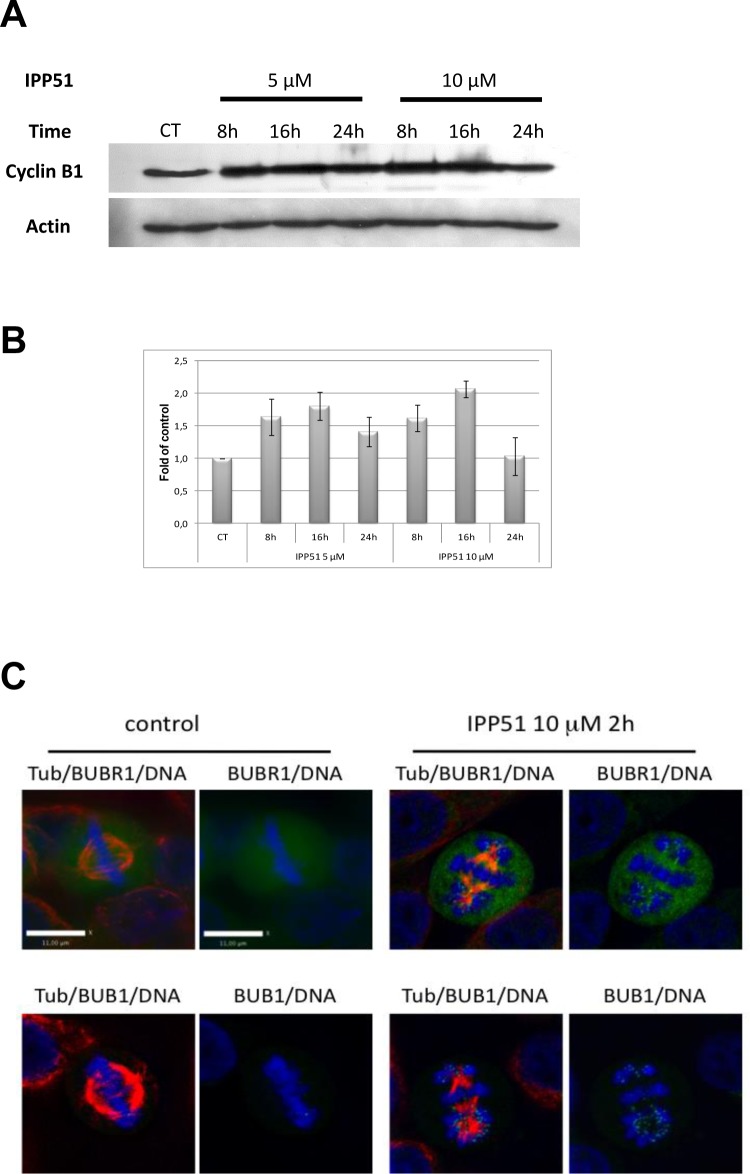
IPP51 induces mitotic checkpoint activation **A.** Western blot analysis of cyclin B1 level in total cell lysates from RT112 cells incubated under indicated conditions. The same blot was probed with an actin antibody for loading control. **B.** The intensity of individual cyclin B1 signal was quantified by densitometry normalizing to that of the control, whose level was arbitrary set to 1. The results shown are the means ± SEM of four independent experiments. **C.** HeLa cells control or treated with 10 μM of IPP51 were fixed and stained for tubulin (red), BubR1 or Bub1 (green) and DNA (blue). Scale bar, 11 μm.

To determine the molecular changes caused by IPP51 that are involved in cell cycle arrest during mitosis, we analyzed the expression of cyclin B1, a key mitotic regulator of cdk1 activity that accumulates progressively during interphase before being degraded at the metaphase/anaphase transition [47]. Western blotting of RT112 cell lysates showed that the level of cyclin B1 was significantly increased under IPP51 treatment (Figure 3A). This accumulation of cyclin B1 was detected from 8 h after the addition of IPP51 for both concentrations tested (5 μM and 10 μM), and 16 h after the addition of 10 μM IPP51, cyclin B1 expression was two-fold greater than that in untreated cells (Figure 3B). This effect was then progressively attenuated, as the cyclin B1 expression returned to its normal level after 24 h of treatment (Figure 3B).

BubR1 and Bub1 are protein kinases of the spindle checkpoint complex that accumulate on kinetochores of mitotic cells during pre-metaphase where they monitor the proper microtubule kinetochore interactions; at metaphase, their disappearance from the kinetochrores triggers the transition from metaphase to anaphase [48, 49]. Visualization of BubR1 and Bub1 in mitotic HeLa cells showed that BubR1 accumulates on chromosomes during prometaphase but is no longer associated with DNA at metaphase (Figure 3C). In contrast, some BubR1 spots were still visible in IPP51-treated cells arrested at metaphase. Bub1 accumulation on kinetochores of misaligned chromosomes in IPP51-treated cells arrested at metaphase was much more obvious than that of BubR1 (Figure 3C). In contrast, in untreated cells (control) Bub1 was associated with chromosomes during prometaphase but not metaphase.

### IPP51 affects tubulin assembly *in vitro* and ***in cellulo***

**Figure 4:**
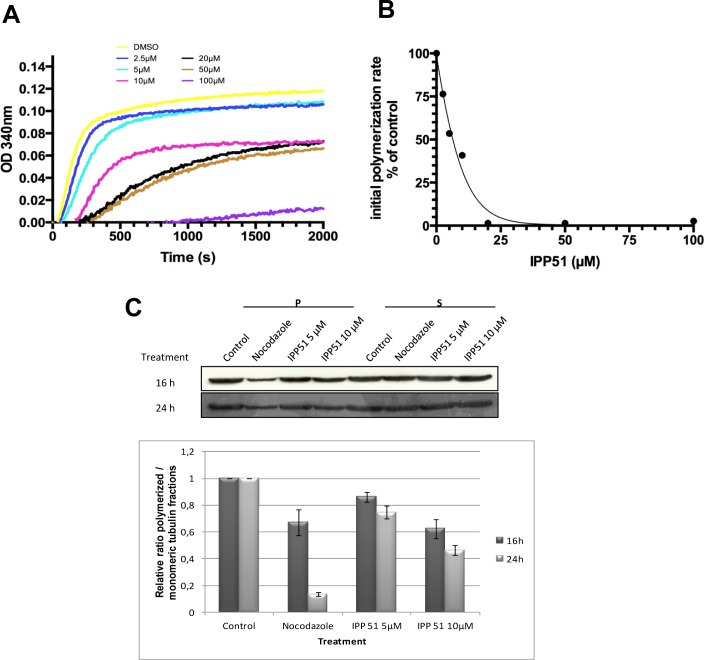
IPP51 inhibits tubulin polymerization *in vitro* and in RT112 cells **A.** Tubulin polymerization in the presence of vehicle (DMSO) or a range of IPP51 concentrations. **B.** The initial rates of tubulin polymerization were calculated from the slopes of the linear elongation phases before reaching microtubule polymer mass steady state by linear regression at each concentration. The percentage of inhibition in the initial rates of polymerization compared to the control was calculated and data points were fitted using EC50 shift equation. **C.** *Upper*. RT112 cells were incubated with nocodazole or IPP51 for 16 or 24 h. Fractions containing polymerized (pellet, P) and soluble (supernatant, S) tubulin were then separated by centrifugation and analyzed by western blot. *Bottom*. The intensity of tubulin bands were quantified by densitometry normalizing to that of the control, whose level was arbitrary set to 1. The results shown are the means ± SEM of three independent experiments.

The above observation that IPP51 affects the cellular organization of microtubules prompted us to examine its possible effect on tubulin polymerization. Tubulin assembly kinetics was studied using an *in vitro* turbidity assay with purified tubulin in the presence or absence of IPP51 [50]. The time course of turbidity variation generated a sigmoid curve, with steady state being reached at 40 minutes with our experimental conditions (Figure 4A). When IPP51 was added, the rate and overall extent of tubulin assembly decreased in a dose-dependent manner. The addition of 10 μM IPP51 resulted in 1.75 times less polymerized tubulin than in the control. Although, there was a progressive delay in the time of onset and a progressive decrease in the initial rate of polymerization with increasing concentrations of IPP51 (10-50 μM), the plateau values for mass steady state were similar, but not identical, for all concentrations. However, at 100 μM IPP51, polymer mass was decreased by 89% compared to the control. At each concentration, the initial bulk rate of tubulin polymerization was calculated from the slope of the linear elongation phase, before microtubule polymer mass steady state was reached, by linear regression. As shown in Figure 4B, IPP51 inhibited microtubule bulk elongation rates, with an IC_50_ of 4.89 μM.

To determine if this inhibitory effect of IPP51 on tubulin polymerization could be detected *in cellulo*, we quantified polymerized tubulin in cells by western blotting. After incubation with IPP51 for 12 or 24 h, cells were extracted with a microtubule-stabilizing buffer to separate polymerized tubulin from free tubulin. As expected, treatment of cells with nocodazole, a known microtubule polymerization inhibitor, led to an increase in the free tubulin fraction (Figure 4C). IPP51 had the same effect, albeit to a lesser extent. Simultaneously, the free tubulin fraction increased and the polymerized tubulin fraction decreased in a time and concentration-dependent manner. The relatively low effect of IPP51 on the level of polymerized tubulin compared to that of nocodazole is in agreement with the observation by indirect immunofluorescence microscopy of no notable changes in microtubule cytoskeleton organization in interphase cells exposed to the same IPP51 concentrations as used in the western blotting experiments (Figure 2B).

Altogether, our results strongly suggest that IPP51 binds directly to tubulin resulting in inhibition of its polymerization both *in vitro* and *in cellulo*.

### IPP51 binds tubulin at the colchicine binding site

**Figure 5:**
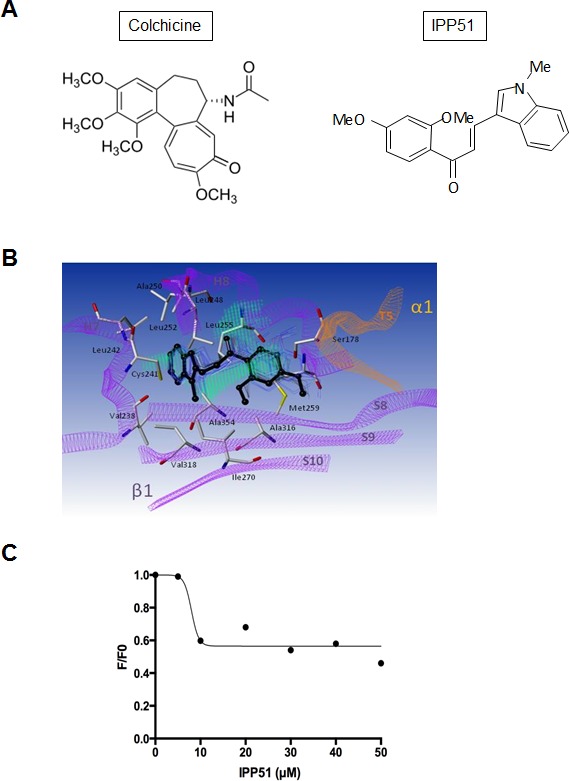
IPP51 binds at the colchicine binding domain of tubulin **A.** Chemical structures of colchicine compared to IPP51. **B.** The β-subunit of tubulin is represented as purple ribbons whereas the α-subunit of the same heterodimer is represented as orange ribbons. Residues interacting with the ligand are in sticks and labeled in black whereas IPP51 best-ranked pose (GOLD score 56.9) according to docking calculation is represented in black ball and stick. Snapshots extracted from MD simulation are also reported as blue lines indicating the stability of the complex (ΔGMM-GBSA −37.2 +/− 1.8 kcal/mol). Hydrophobic fitting points calculated by using the GOLD-MLP approach are visualized as green dots. **C.** Competitive binding assay of colchicine. The decreased stable fluorescence ratio of colchicine (F) at each IPP51 concentration to the original fluorescence intensity of tubulin (F0) in the absence of IPP51 was plotted and fitted.

Numerous chalcone compounds that inhibit the assembly of tubulin into microtubules bind to the colchicine-binding site of tubulin [51]. Colchicine (Figure 5A) binding to tubulin causes tubulin to acquire a straight structure, which prevents it from polymerizing into microtubules. To determine whether IP551 can bind to the colchicine-binding site of tubulin, a computational study was performed. The crystallographic structure of tubulin in complex with DAMA colchicine from Bos Taurus (99% identity with human tubulin for which no X-ray information is available, Supplementary Figure S1) was used as a starting point for molecular docking calculations [52]. Due to the hydrophobic properties of the colchicine binding domain (lipophilic index of 11), a GOLD-MLP approach was used for the molecular docking calculations [39, 40]. Hydrophobicity was described by the molecular lipophilicity potential (MLP), a molecular interaction field that relies on an atomic fragmental system based on 1-octanol/water experimental partition coefficients. The docking protocol was able to position the best-ranked DAMA colchicine in the niche localized between the two α and β tubulin subunits with a RMSD value of 1.0 Å with respect to crystallographic information (Supplementary Figure S2). Following this successful strategy, IPP51 was docked in the colchicine binding site of β-tubulin (Figure 5B). The best ranked pose for the indol group of IPP51 was found to be trapped between helices H7 (Val238, Cys241, and Leu242) and H8 (Leu248, Ala250, and Leu255), whereas the aromatic moiety of IPP51 formed hydrophobic interactions with Leu242 (H7), Ala316 and Ala354 (S8-S10). No hydrogen bonds were observed between IPP51 and the protein. MD simulation for 1 ns confirmed the stability of the complex in explicit water molecules, with a RMSD value of 0.9 +/− 0.2 for the ligand atoms. Moreover, MM-GBSA thermodynamic calculations confirmed a favorable binding mode for IP551, with a ΔG_MM-GBSA_ value of −37.2 +/− 1.8 kcal/mol.

The docking results predicted that IPP51 shares the same tubulin binding site as colchicine. Affinity-induced fluorescence enhancement of colchicine with tubulin (excitation, 362 nm; emission, 435 nm) has been previously demonstrated [41]. To confirm that IPP51 interacts with the colchicine-binding site of tubulin *in vitro*, we measured the fluorescence emission of the colchicine-tubulin complex formed in the presence of increasing concentrations of IPP51. Indeed, when tubulin was added to the mixtures of colchicine and IPP51, significant inhibition (close to 50%) of fluorescence development occurred at IPP51 concentrations higher than 10 μM (Figure 5C), suggesting that the IPP51 can compete, albeit partially, with colchicine binding to tubulin. These data confirmed our docking results and led us to conclude that IPP51 interacts with tubulin at the colchicine binding site, leading to an inhibition of microtubule assembly.

### IPP51 exhibits anti-angiogenic properties

**Figure 6:**
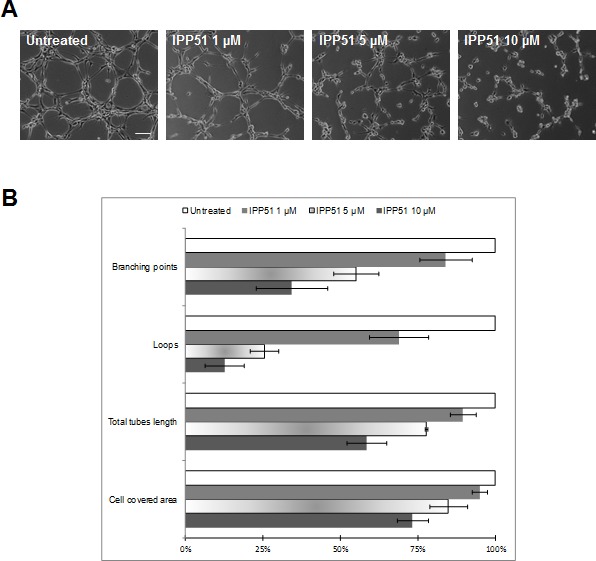
IPP51 inhibits capillary-like tubes formation HUVEC cells were seeded on Matrigel with 1, 5 or 10 μM of IPP51 or without drug (untreated). Network was observed 5 h later with a phase-contrast microscope. **A.** Representative pictures of each culture condition (X10 magnification, *Bar = 100 μm*). **B.** Quantitative analysis of IPP51 effects on dimension and topology of endothelial cells network. Data represent mean ± SD of two independent experiments.

Recent studies have shown that most microtubule-binding agents have anti-angiogenic and/or vascular-disrupting activities [53]. Moreover, flavonoids and chalcones have been shown to inhibit angiogenesis through different mechanisms, such as regulation of VEGF expression [54]. Thus, we evaluated the effect of IPP51 on capillary-like tube formation by HUVECs (human umbilical vein endothelial cells) plated on Matrigel. In control conditions, after 5 h, we observed that Matrigel provides a good environment for the organization of endothelial cells into a network of tubes (Figure 6A). When IPP51 was added at 5 or 10 μM at the time of cell seeding, there was a strong inhibition of tube formation (Figure 6A). Quantification of the characteristics of the capillary-like network of HUVECs showed that IPP51 decreased both dimensions of that network (total area covered by cells and total tube length) and its topology in a concentration dependent manner (Figure 6B). This indicated that IPP51 is a potent anti-angiogenic compound.

### IPP51 inhibits tumor growth of bladder cancer cell xenografts *in vivo*

**Figure 7:**
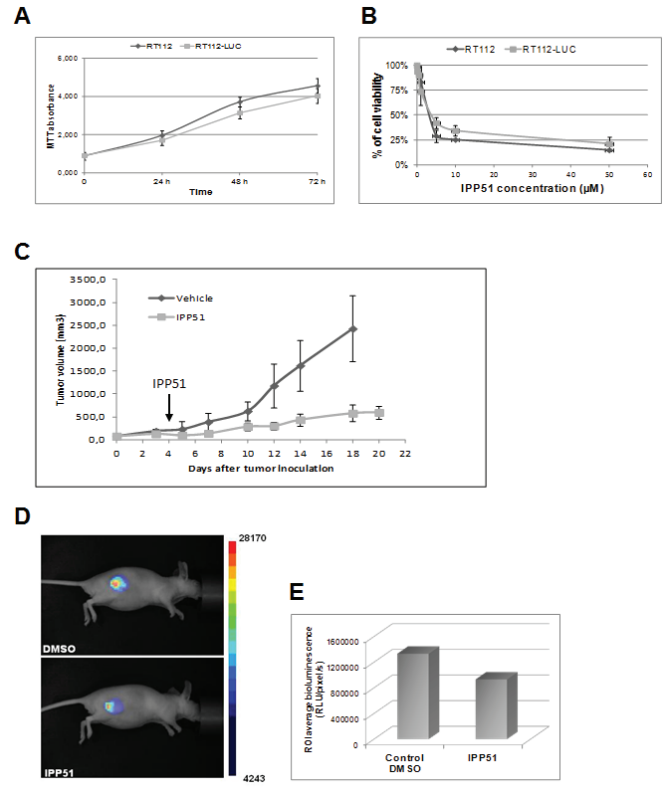
*In vivo* activity of IPP51 in mice bearing RT112 xenografts **A.** The luciferase gene did not affect the growth RT112 cells behavior. RT112 and RT112-LUC cells were grown for four days and their proliferation was evaluated by a MTT assay. **B.** The effect of IPP51 on RT112 and RT112-LUC cells growth was studied by a MTT test. The cell viability is expressed as the percentage of the untreated control cells. The data are the mean of triplicates ± SD of three independent experiments. **C.** After tumor formation, mice were treated with IPP51 or DMSO (control) by intratumoral injections every two days up to twenty days. The tumor volumes were measured by caliper every three days. Note that at twenty days result is not shown for the control because the tumors size became too important and the mice had to be sacrificed. Each point represents mean tumor volume for three animals in each group. These data are representative of results obtained in two independent experiments. **D.** Bioluminescence images of mice DMSO or IPP51 treated obtained at the end of the experiment (twenty days). **E.** Measurement of the bioluminescence intensities, expressed as the number of relative light units (RLU) per pixel per second for a specified region of interest (ROI).

We examined whether IPP51 inhibits tumor growth in an *in vivo* bladder cancer xenograft model. We chose to implant luciferase-transfected RT112 cells for this model, which allowed us to directly monitor tumor development by bioluminescence [55]. As a control, we first checked whether the luciferase gene alone had a negative influence on cell growth *in vitro*. The proliferative rates of the RT112 parental cell line and a stably expressing luciferase clone were assessed by MTT assay. As shown in Figure 7A, RT112-LUC cells (luciferase stable transfectants of RT112 cells) displayed similar growth dynamics to those of parental RT112 cells. Next, we examined whether IPP51 has the same cytotoxic effect against RT112-LUC cells and parental RT112 cells (Figure 7B). The calculated IC_50_ of IPP51 for RT112-LUC cells was 4.1 ± 0.22 μM, which was very close to that for RT112 parental cells (5 ± 0.31 μM). These results justified the use of RT112-LUC cells instead of RT112 parental cells for our *in vivo* experiments. Cells were injected subcutaneously into the right flanks of nude mice, and when tumors were palpable and detectable by bioluminescence imaging, the animals were randomized into two cohorts of four animals each: one group received IPP51 and the other group received vehicle as a control (1.5% DMSO in PBS). The treated mice received 50 μg of IPP51 by intratumoral injection every two days for up to twenty days. No signs of redness or inflammation were observed at the IPP51 injection site. As shown in Figure 7C, the tumor volume of the control group increased to 2423 ± 719 mm^3^ at the end of experiment. It should be noted that we were forced to sacrifice the animals at day 18 because the tumors had become too large. In contrast, mice treated with IPP51 remained healthy without signs of toxicity. The tumor growth of the IPP51-treated animals was significantly inhibited; the tumors grew to only eight times their starting volumes in the IPP51-treated animals in comparison to thirty-two times their starting volumes in the control-treated animals. After twenty days, the tumor volume the IPP51-treated animals was 595 ± 146 mm^3^. The results were obtained by monitoring tumor progression by traditional caliper measurement and were confirmed by bioluminescent imaging (Figures 7D-7E). At twenty days (sixteen days of treatment), the volume of the IPP51-treated tumors was reduced by approximately thirty-times that of the control group.

**Figure 8:**
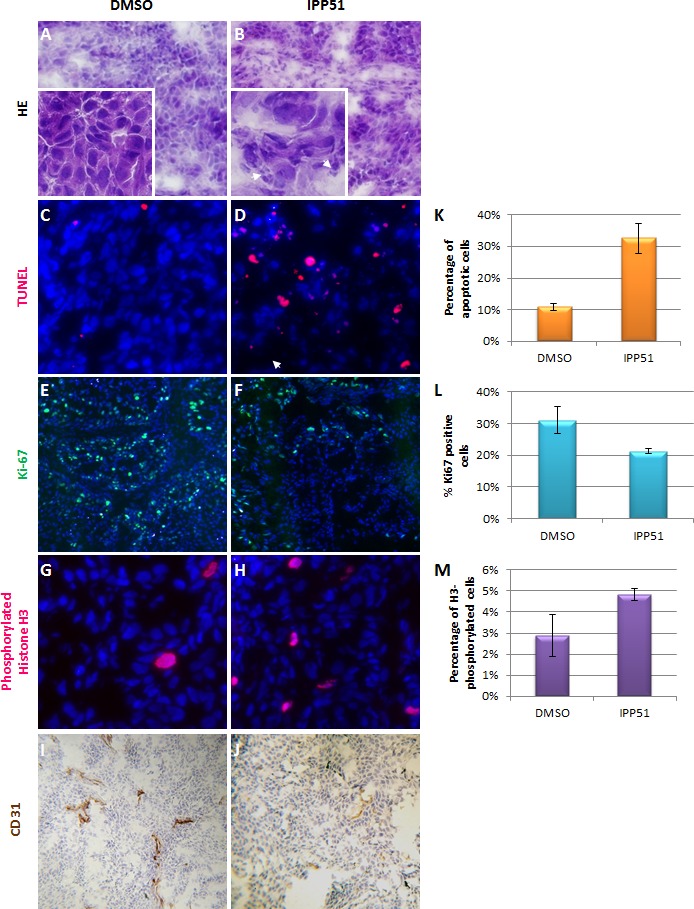
IPP51 induces cell proliferation inhibition, mitotic arrest, apoptosis and angiogenesis inhibition *in vivo* **A.**-**J.** Representative images of sections from tumor xenografts obtained from control mice (left panel) or IPP51 treated mice (right panel). **A.** and **B.** correspond to a H & E staining. Arrowheads show pyknotic (densely stained) and fragmented nuclei, indicating cell death. **C.** and **D.** showed result of TUNEL labeling (red) to measure apoptosis. **E.** and **F.** correspond to staining of Ki-67 (green), a proliferation marker. **G.** and **H.** are representative pictures of phosphorylated histone H3 staining (red), a mitotic marker. DNA in **C.**-**H.** sections was counterstained with Hoechst (blue). **I** and **J** were obtained after immunohistochemical labeling of CD31. **K.**-**M.** Tumor slides from control and IPP51 treated mice were visualized under microscope and TUNEL, Ki67 and phosphorylated histone H3 positive cells were quantified. Two slides per sample were analyzed, counting 500 cells per slide in randomly selected fields. Data represent the arithmetic mean ± SEM of 3 mice.

As IPP51 induced a significant reduction in tumor volume, we searched to determine whether this was accompanied by inhibition of cell proliferation and/or apoptosis induction using tumor sections. Figures 8A and 8B showed histological examination of H&E stained sections obtained from tumors excised from control and IPP51-treated mice. Several necrotic foci with pyknotic and fragmented cell nuclei were observed in sections derived from IPP51-injected tumors. This indicated that IPP51 induced cell death. This was further assessed by a TUNEL assay, which showed a significant number of apoptotic nuclei in tumors from IPP51-treated mice (Figures 8C-8D). The percentage of TUNEL-positive cells increased from 11 ± 1% in the control group to 33 ± 5% in the IPP51-treated group (Figure 8K). As observed in RT112 cells in culture, immunohistochemical analysis of Ki-67, a cellular marker of cell proliferation [56], confirmed that IPP51 injection resulted in a decrease in cell proliferation of approximately 1.5-fold in the IPP51-treated xenograft tumors compared to the vehicle-treated xenograft tumors (Figures 8E-8F and 8L). We also measured the mitotic index of RT112-tumor xenografts to confirm the mechanism of action of IPP51 *in vivo*. Immunostaining of phosphorylated histone H3 (Figures 8G-8H), a marker of mitotic chromosome condensation [57], in sections from xenograft tumors showed an increase in sections from IPP51-treated mice (~ 1.7-fold greater than vehicle-treated mice, Figure 8M). It should be noted that only a limited proportion of tumoral cells expressed H3-phosphorylated histone or Ki-67 proteins. This could be due to the proliferation rate paradox in tumors [58]. In fact, low mitotic indices are frequently observed in solid tumors, and it seems that cancer cells do not proliferate quicker than normal cells but, rather, do so in an untimely fashion. This led to the recent hypothesis that microtubule-targeting agents could have effects on cellular processes other than mitosis [59, 60]. Finally, we examined whether IPP51 inhibits tumor angiogenesis. Tumor sections were stained for CD31, a specific and sensitive marker of endothelial cells. As shown in Figures 8I-8J, IPP51 seemed to reduce the number of tumor blood vessels. Altogether, the results of our RT112 tumor xenograft experiments were consistent with those previously observed in cultured cells, demonstrating that IPP51 retained its activity *in vivo*.

## DISCUSSION

IPP51, a chalcone derivative, was initially identified as a potential anticancer agent that kills bladder cancer cells through caspase-dependent apoptosis following cell cycle arrest at the prometaphase stage. While inhibiting proliferation of bladder and cervical cancer cells, IPP51 showed no toxicity against normal urothelial or lung cells, which incited us to further characterize IPP51 and to identify its cellular target(s). First, we observed that the prometaphase arrest caused by IPP51 is associated with aberrant mitotic spindles, accumulation of cyclin B1 and activation of the spindle assembly checkpoint proteins. These findings prompted us to investigate whether IPP51 targets tubulin. Our *in vitro* microtubule polymerization assay suggested that IPP51 is a new inhibitor of microtubule assembly. Furthermore, virtual docking of the IPP51 to tubulin supported the observation that IPP51 competes with colchicine for the same tubulin binding site *in vitro*. Finally, the putative chemotherapeutic potential of IPP51 was confirmed by the observation of reduced tumor growth in a mouse xenograft model.

IPP51 induced a mitotic block associated with aberrant spindle formation and chromosome misalignment. Live cell imaging of HeLa cells after they assembled bipolar spindles demonstrated that the addition of IPP51 results in the disorganization of the bipolar mitotic spindle due to spindle microtubules shortening and splitting from the two spindle poles into multiple microtubule asters with short microtubules. Treated bladder cancer cells also appeared to have an increase in microtubule organizing centers, which may be due to centrosome splitting and/or the inheritance of aberrant centrosome numbers following mitotic failure. Although centrosome amplification is considered oncogenic in the absence of a centrosome clustering mechanism, the increased number of centrosomes leads to massive mitotic catastrophe and inhibition of proliferation [61, 62].

Although the mechanisms by which inhibitors of tubulin induce cell death vary depending on the particular inhibitor and cell type, it seems clear that disruption of the mitotic spindle activates the spindle-assembly checkpoint (SAC), ultimately leading to apoptosis [63]. Indeed, IPP51 induced accumulation of the kinetochore-associated checkpoint proteins Bub1 and BubR1 on the chromosomes of HeLa cells. Their recruitment to the kinetochores has been previously attributed to defects in the kinetochore microtubule interaction and, in particular, in the lack of tension generated by the forces exerted by dynamic kinetochore-microtubules [64-66]. The presence of Bub1 and BubR1 in the kinetochores of treated cells suggests aberrant kinetochore-microtubule dynamics and the loss of tension between sister kinetochores. Thus, IPP51 induced a sustained accumulation of the mitotic checkpoint proteins Bub1 and BubR1 on misaligned chromosomes, indicating that SAC activation occurred. This result is consistent with the observed cyclin B1 accumulation and mitotic arrest [67].

Microtubule dynamics are related to the intrinsic tubulin polymerization properties and are regulated by microtubule-associated proteins. In light scattering experiments of MAP-free tubulin assembly, IPP51 inhibited the nucleation time and the bulk microtubule elongation rate in a concentration-dependent manner. The observed IC_50_ value of IPP51 in the microtubule elongation phase of purified tubulin was approximately 5 μM, which coincided with our previous results that 5 μM IPP51 was sufficient to induce 50% loss of RT112 cell viability. Although the concentrations of the inhibitor used in the current study were sufficient to provoke obvious microtubule spindle defects, at interphase, the microtubule cytoskeleton was not affected, and the polymer mass was only slightly diminished. It is well accepted that microtubules in interphase tissue culture cells turnover slower than mitotic microtubules (reviewed in [68, 69]. Therefore, mitotic microtubules may be more prone to inhibition by IPP51 or by any other microtubule-inhibiting agent. On the other hand, recent studies have shown that microtubule-targeting agents could have effects on cellular processes other than mitosis [59, 60]. A quantitative analysis of microtubule dynamics *in cellulo* in the presence of the inhibitor would be valuable for determining, in a more detailed mechanistic manner, the inhibitory properties of IPP51 in interphase and mitotic cells. The challenge now is to decipher which interphase processes are more susceptible in tumor cells following interphase-microtubule perturbations by IPP51 than in normal cells. Such studies may explain the clinical efficacy of anti-mitotic strategies, despite the fact that low mitotic indices are observed in solid tumors.

Microtubule-targeting drugs are often classified into two main groups, namely microtubule-stabilizing agents and microtubule-destabilizing agents. The second class of molecules often target the vinca, or the colchicine-binding site, of tubulin [70]. These molecules act by inhibiting tubulin polymerization and inducing mitotic arrest, eventually leading to apoptosis. Microtubule-depolymerizing agents induce spindle formation defects, characterized by the formation of paracrystalline microtubule arrays with vinca alkaloids [71]. The effect of IPP51 on microtubule polymerization and mitotic spindle formation most closely resembles that of colchicine. Indeed, *in silico* modeling predicted that the site of interaction of IPP51 with tubulin coincides with the previously structurally characterized colchicine binding site located at the interface between the α and β monomers of the same heterodimer. Our virtual docking protocol predicted that the indol group of IPP51 is trapped between helices H7 (Val 238, Cys 241, and Leu 242) and H8 (Leu 248, Ala 250, and Leu 255), whereas the aromatic moiety may be involved in hydrophobic interactions with the Leu242 (H7), Ala 316 and Ala 354 (S8-S10) residues of β tubulin. No hydrogen bonds were observed between IPP51 and the protein. Furthermore, our *in vitro* competition assays for colchicine binding to tubulin in the presence of IPP51 showed that IPP51 could partially displace colchicine from tubulin, indicating that IPP51 and colchicine target the same tubulin binding-site. The failure of IPP51 to fully inhibit colchicine binding to tubulin dimers may indicate differences in the tubulin-ligand binding reaction. Colchicine is known to bind to the tubulin dimer through a biphasic reaction characterized by the formation of a fast and reversible pre-equilibrium complex followed by many conformational changes in tubulin, which results in a poorly reversible tubulin-colchicine complex (reviewed in [72]. The fact that IPP51 fails to fully displace colchicine at excess concentrations relative to colchicine, may be due to the ability of IPP51 to only compete for the first pre-equilibrium tubulin-colchicine complex (with a calculated IC_50_ of 7.29 μM) and not for the second binding phase, which results in the nearly irreversible final tubulin-colchicine complex.

Although colchicine was the first discovered tubulin destabilizing agent that was able to inhibit mitosis, its use as a chemotherapeutic agent is hampered by its low therapeutic index due to its severe toxicity to normal tissues [29]. However, it has been shown that drugs targeting the colchicine-binding site on tubulin circumvent class III β-tubulin resistance, which is one of major limitations of the clinical use of microtubule-targeting agents [73]. Therefore, IPP51 could have a clinical advantage in tumors overexpressing the β III-tubulin isoform.

Recently, tubulin binding agents received renewed interest as anti-angiogenic drugs [2, 53]. Angiogenesis is a key step in tumor progression and is a complex process through which new blood vessels are formed from the pre-existing vasculature [74]. The endothelial cell cytoskeleton plays an essential role in this highly regulated process, which may explain why microtubule-targeting agents show anti-angiogenic properties. Indeed, we found that IPP51 inhibits capillary-like tube formation by HUVEC cells embedded in Matrigel. Further studies would determine how IPP51 interferes with the microtubule dynamics of endothelial cells. We will also study if IPP51 can inhibit endothelial cell proliferation or induce endothelial cell apoptosis, which could be an advantage for disrupting formed tumor blood vessels. Finally, our preliminary studies on tumor xenografts indicated that IPP51 maintains its anti-angiogenic activity *in vivo*. However, complementary studies will be necessary to quantify this effect and to determine whether IPP51 affects tumor vessel structure. In summary, this study identifies tubulin as the molecular target of IPP51. This easily synthesized chalcone derivative binds to the colchicine binding site and inhibits tubulin polymerization both *in vitro* and *in vivo*. IPP51 targets the mitotic spindle microtubules, suggesting that its induction of apoptotic death can be attributed to mitotic arrest caused by perturbation of spindle assembly and activation of the spindle assembly checkpoint. We have not yet analyzed whether the dead cells were arrested in mitosis or in G1 after slippage from mitosis. Nevertheless, our studies in a mouse model with xenografts of human bladder tumor cells showed that tumor shrinkage is associated with lower cell proliferation linked to a higher mitotic index and apoptotic cell death at IPP51 dosages of 50 μg every two days. Interestingly, IPP51 exhibited significant reduction of tumor growth with no signs of toxicity, which indicates that IPP51 has chemotherapeutic potential. No drug currently approved for clinical use targets the colchicine-binding site of tubulin, suggesting that IPP51 is an interesting drug candidate for further studies.

## MATERIALS AND METHODS

### Materials

Antibodies against β-tubulin (clone TUB 2.1), γ-tubulin and actin were purchased from Sigma (Lyon, France). Rabbit anti-Bub1 and anti-BubR1 antibodies were a kind gift from T. Yen (Fox Chase Cancer Institute, Philadelphia). Cyclin B1 antibody was purchased from Epitomics (Burlingame, USA). Antibodies against Ki-67 and Ser 10-phosphorylated histone H3 were purchased from Abcam (Cambridge, UK) and Cell Signaling Technology (Danvers, USA) respectively. Anti-CD31 antibody (clone MEC 13.3) was obtained from BD Pharmingen (Le Pont de Claix, France). Antibody Alexa Fluor 488 and 568-conjugated secondary antibodies were obtained from Life Technologies (Paisley, UK).

### Cell culture

Cells were cultured as described in Supplemental Materials and Methods.

### Chalcone synthesis

1-(2,4-Dimethoxyphenyl)-3-(1-methylindolyl) propenone, namely IPP51 was prepared and characterized according to our previous report [19]. It should be highlighted that the IPP51 was obtained exclusively as the E isomer as clearly and unambiguously determined by 1H NMR. The Z isomer formation has never been observed. Therefore, through all our study, the E isomer was used including for the docking studies.

### Cell proliferation assay

Inhibition of cell proliferation was measured by a MTT assay as previously described [20].

### Indirect immunofluorescence

RT112 cells were plated on coverslips the day before treatment with IPP51 for 16 h or 24 h. For β-tubulin visualization, cells were fixed for 15 min in 4% paraformaldehyde and then permeabilized for 10 min with 0.2% Triton X-100 in PBS. For γ-tubulin labeling, fixation was performed in methanol at −20°C for 10 min. After incubation with primary antibodies against tubulin followed by incubation with Alexa Fluor 488-conjugated secondary antibodies, DNA was counterstained with Hoechst (Euromedex, Souffelweyersheim, France). Coverslips were mounted on glass slides using the mounting liquid Fluorsave™ Reagent (Calbiochem, Merck KGaA, Darmstadt, Germany). Cells were observed with an Axiovert 135 inverted microscope (Zeiss, Le Pecq, France). Images were acquired using a black and white XM10 camera (Olympus France SAS, Rungis, France) and the Cell Sens software (Olympus).

For detection of Bub1 and BubR1 proteins, HeLa cells were grown on poly-Dlysine-coated glass coverslips. Cells were fixed with 1% paraformaldehyde-PBS for 20 min, washed for 5 min with PBS, permeabilized with 0.2% Triton X-100 in PBS for 3 min, and washed again for 5 min with PBS. Then, the cells were processed with primary and secondary antibodies. DNA was detected with DAPI in the VECTASHIELD mounting medium (Vector Laboratories Inc, Burlingame, CA).

### Western blotting

Adherent and suspended cells were harvested, washed twice with PBS and lysed in RIPA buffer containing a protease inhibitor cocktail (Complete, Roche, Meylan, France). After 45 min on ice, the lysates were centrifuged at 20,000 x *g* for 15 minutes, and soluble proteins were quantitated using the QuantiPro BCA kit (Sigma). Samples (50 μg of total protein) were resolved by 10% SDS-PAGE and transferred onto nitrocellulose membranes. The membranes were blocked with 5% nonfat dry milk, incubated overnight at 4°C with cyclin B1 or actin antibodies and then incubated with goat anti-rabbit or anti-mouse HRP-conjugated antibodies for 45 min at room temperature. The membranes were developed with ECL substrate (Perkin Elmer, Courtaboeuf, France).

### Extraction of soluble and polymerized tubulin fractions

Extraction was done using a modification of the protocol described by Moon *et al*. [37]. After treatment with drugs for 16 h or 24 h, medium containing cells in suspension was recovered and pooled with adherent cells scraped in PBS pre-warmed at 37°C. After centrifugation 5 min at 400 x g and a wash with PBS, cells were extracted for 5 min with pre-warmed at 37°C microtubule-stabilizing buffer (0.1 M PIPES pH 6.9, 14.5% glycerol, 0.5% Triton X-100, 20 mM EGTA and 5 mM MgCl_2_) containing Complete (protease inhibitor cocktail from Roche) and 10 ng/ml paclitaxel (Sigma). After centrifugation at 20 000 x g for 10 min at 25°C, supernatants containing soluble fractions were transferred to a new tube, while polymerized fractions in pellets were recovered by incubation in RIPA buffer for 45 min on ice followed by centrifugation for 10 min at 20 000 x g. Twenty microliters of soluble and polymerized fractions separately mixed with 4X Laemmli buffer were analyzed by western blotting realized as mentioned above.

### Microtubule polymerization assays

Microtubule polymerization assays were performed in 96-well half-area μclear plates using a 96-well photometer (TECAN) to measure absorbance at a wavelength of 340 nm. All experiments were performed at the same time using the same buffers and MAP-free tubulin [38] from the same preparation to minimize variations originating from different experimental conditions. The final test volume was 50 L and was prepared from solutions kept at 4°C. The final tubulin concentration was 20 μM in PEM buffer (100 mM PIPES, 1 mM MgCl_2_, 1 mM EGTA, and 1 mM GTP), and tubulin was supplemented with increasing concentrations of inhibitor in DMSO; the DMSO concentration was adjusted to be equal in all wells (10%). The polymerization of microtubules was followed at 340 nm at 37°C. Data were fitted using GRAPHPAD PRISM 6 software.

### Molecular modeling

Docking calculations were carried out using GOLD version 5.1 (CCDC, Cambridge, UK) and the MLP-filter approach [39, 40]. Details of the docking studies are described in Supplemental *Materials and Methods*.

### Colchicine-tubulin binding competition assay

Fluorescence measurements of the binding of colchicine to tubulin were performed with a MOS-250 optical system (Bio-Logic S. A, Claix, France), as described by Bhattacharyya and Wolff [41]. Excitation was at 353 nm, and emission was read at 430 nm. Measurements were performed at 37°C in a 1 × 1-cm fluorescence cuvette under continuous stirring. Tubulin (1 μM final concentration) was added to 5 μM colchicine (from 2.5 M stock solution in DMSO) in the absence or presence of increasing IPP51 concentrations. Changes in the ratio of F/Fo, where F is the fluorescence of the colchicine-tubulin complex formed in the presence of a given IPP51 after 1 h and Fo is the fluorescence of an equal concentration of colchicine in excess tubulin, were plotted as a function of the inhibitor concentration. Data points were fitted using GRAPHPAD PRISM 6 software.

### *In vitro* angiogenesis assay

An initial layer of Matrigel matrix (Corning, Amsterdam, The Netherlands) of 150 μl per well in a 24-well plate was allowed to polymerize for 30 min at 37°C. Then, a second layer of 150 μl Matrigel per well was applied and allowed to polymerize for 30 min at 37°C. The gel was overlaid with 8 × 10^4^ HUVEC cells in 1 ml of complete medium containing a series of the same concentrations of IPP51 or DMSO. After 5 h at 37°C, phase contrast images were recorded and analyzed using the automated WimTube image analysis tool (Wimasis GmbH, Munich, Germany). Data were expressed as percentages in comparison to cells treated with an equal volume of vehicle alone (DMSO). All values presented here are the means of two independent experiments. For each well, 3 distinct fields of view were analyzed and averaged.

### Tumor xenograft studies

All animal experiments were conducted in adherence to the Principles of Laboratory Animal Care (National Institutes of Health publication no 86-23, revised 1985) and approved by the regional ethics committee (Reference number for animal experiments: 91_IAB-U823 MK-04; Comité d'éthique en experimentation animale de Grenoble: Com-Eth, amended by the Comité National de Réflexion Ethique sur l'Expérimentation animale (N°.12)).

Female athymic NMRI nude mice purchased from Janvier (Le Genest Saint Isle, France) at 6 to 8 weeks of age were maintained under specific pathogen-free conditions. Xenografts were established by injecting 5 × 10^6^ of luciferase-expressing cells subcutaneously into the right flanks of the mice. On day 4, tumor size was measured, the animals were randomized into 2 groups (*n* = 4 per group) and drug treatment was initiated. The control group was treated with vehicle (1.5% DMSO in PBS). The drug (or vehicle) treatment was performed for 20 days. Tumor size was measured twice a week. The tumoral volume was calculated as l^2^ x L x 0.52.

### Histological analyses

At the end of the *in vivo* experiment, the mice were sacrificed, and the subcutaneous tumors were excised and frozen. Frozen sections (8 μm) of the tumors were made for further analysis. Hematoxylin / eosin staining was performed as previously described [42]. For immunostaining of proliferation and apoptosis markers, frozen sections were fixed with 4% paraformaldehyde, permeabilized in PBS / 0.2% Triton-X100, and then incubated with antibodies. Immunohistochemical staining of CD31 was performed as previously described [43]. For quantification of apoptosis, TUNEL labeling was performed according to the manufacturer's instructions (Roche).

## SUPPLEMENTARY MATERIALS FIGURES AND MOVIES






