# Supplementary material for: IPP51, a chalcone acting as a microtubule inhibitor with in vivo antitumor activity against bladder carcinoma
Source: Oncotarget. 2015 May 15;6(16):14669–86. doi: 10.18632/oncotarget.4144 (PMC4546496; doi:10.18632/oncotarget.4144)
Supplement: Supplementary file 3 [file oncotarget-06-14669-s003.pdf]

IPP51, a chalcone acting as a microtubule inhibitor with *in vivo* antitumor activity against bladder carcinoma

Supplementary Material

Supplemental Information contains:

-Supplementary Materials & Methods

-Supplementary Figures S1 and S2, which are related to Figure 5

-Legends of supplementary Movie S1 and Movie S2

SUPPLEMENTARY MATERIALS & METHODS

Cell culture.

Human bladder cancer cell line RT112 was obtained from Cell Lines Service (Eppelheim, Germany). RT112 cells were cultured in RPMI 1640 medium supplemented with 10% (v/v) fetal calf serum (FCS) and Glutamax (Invitrogen Life Technologies, Paisley, UK). HeLa cells (American Type Culture collection, No. CCL2) were grown in Glutamax DMEM supplemented with 10% (v/v) FCS, penicillin (2.5 U/ml) and streptomycin (2.5 µg/ml).Human telomerase reverse transcriptase -immortalised normal human urothelial cells (TERT-NHU cells) were kindly provided by M.A. Knowles (Cancer Research UK Clinical Centre, St James's University Hospital, Leeds, UK). These cells have normal karyotype and are non-tumorigenic (Chapman, 2006). TERT-NHUC were plated onto Primaria culture vessels (BD Biosciences, Le Pont de Claix, France) and maintained in KSFM (Life Technologies SAS, Saint Aubin, France) supplemented with the supplied bovine pituitary extract and epidermal growth factor plus 30 ng/ml cholera toxin (Sigma, Lyon, France). All cells were maintained at 37°C in a 5% CO_2-humidified atmosphere. IMR-90 cells were obtained from Coriell Cell Repositories (Camden, New Jersey, USA) and were cultured in DMEM high glucose medium (Invitrogen Life Technologies) supplemented with Glutamax, 10% FCS, penicillin (2.5 U/ml) and streptomycin (2.5 µg/ml). Human umbilical vein endothelial cells (HUVEC) were purchased from Lonza (Basel, Switzerland) and cultured in EGM-2 Basal Medium supplemented with EGM-2 SingleQuot Kit supply & Growth Factor (Lonza). Cells are used until the third passage.

Live cell imaging and video microscopy.

For time-lapse microscopy, HeLa cells expressing GFP-tubulin were plated in glass-bottom dishes then placed inside a video microscopy platform equipped with an incubator enabling the regulation of the temperature. Just before recording, the cell growth medium was replaced with the CO_2 independent growth medium DMEM/F12 with15 mM HEPES (Invitrogen Life Technologies) supplemented with 10% serum with or without 10 µM IPP51. Time-lapse Z series images (Z = 3) were collected with an inverted Olympus IX81 epifluorescence motorized microscope equipped with an environmental control chamber. The microscope was equipped with a motorized piezo stage (Ludl Electronic Products, Hawthorne, USA) and a Retiga- SRV CCD camera (QImaging, Surrey, Canada) driven by VOLOCITY software (Improvision, PerkinElmer, Waltham, USA) with a binning of 1, using a PlanApo 60xNA 1.42 objective (Olympus, Rungis, France). Images were acquired every 5 min and the acquisition time was 53 ms. For each Z series, the best focus images was chosen before the reconstitution of the movie.

Molecular modeling.

The crystallographic structure of tubulin from Bos Taurus (99% identity with the human tubulin) in complex with DAMA colchicine was retrieved from the Protein Data Bank (PDB ID: 1SA0) [(1)]. The receptor was prepared by using the Biopolymer tool of Sybyl-X2.0 (Tripos Inc.,

St. Louis, MO, USA): co-crystallized ligands and water molecules were extracted, missing side chain residues and hydrogen atoms were added, Tripos atom and bond types were assigned to both protein and extracted ligand [(2)]. The rigid binding site was defined by a 6 Å radius sphere around the co-crystallized ligand. 100 docking solutions were generated by using 100'000 GOLD Genetic Algorithm iterations (Preset option), ranked according to the GOLD score. Root-mean-square-deviation RMSD values between the docking solutions and the crystallized ligand of reference were calculated. Moreover, a RMSD clustering analysis was performed. By following the same approach, IPP51 was docked to the protein by applying the methodology described above. The best ranked pose in complex with tubulin was then used for carrying out 1ns Molecular Dynamics (MD) simulation with Amber 12 [(3)] by using Gaff and Amberff99SB force fields for the ligand and the protein, respectively. The partial charges of ligand were assigned with the AM1-BCC method, compatible with the Amber force field. The complex was then embedded in an octahedral box of 10 Å TIP3P water molecules neutralized by Na^+ ions and minimized along 1000 steps with restraints on the solute using the *pmemd* module of Amber 12. Complexes were then free to relax along further 2500 minimization steps. 100 ps of heating at constant volume (from 10 K to 300 K) with weak restraints on the solute (10.0 kcal/mol/Å) was applied, followed by 750 ps MD at constant pressure conditions (1 atm) reaching an equilibrium state. Trajectories were then collected each 2 ps along 1 ns of MD production phase. The Particle-Mesh-Ewald algorithm (PME) with a cut-off of 8 Å was used to treat the longrange electrostatic effects [(4)]. The SHAKE algorithm was also used to constrain the bonds connecting hydrogen atoms [(5)]. A MM-GBSA single trajectory approach was then performed on 1000 snapshots extracted every 1 ps for free energies of binding ($\Delta G_{MM\text{-}GBSA}$) estimation [(3)].

[1]Ravelli RB, Gigant B, Curmi PA, et al. Insight into tubulin regulation from a complex with colchicine and a stathmin-like domain. Nature 2004; 428: 198-202.

[2]Clark M, Cramer RD, Van Opdenbosch N. Validation of the general purpose tripos 5.2 force field. Journal of Computational Chemistry 1989; 10: 982-1012.

[3]Case D, Darden T, TE CI, CL S, J W, al., Amber 12. University of California, San Francisco 2012.

[4]Darden T, York D, Pedersen L. Particle mesh Ewald: An Nâ‹…log(N) method for Ewald sums in large systems. The Journal of Chemical Physics 1993; 98: 10089-92.

[5]Ryckaert J-P, Ciccotti G, Berendsen HJC. Numerical integration of the cartesian equations of motion of a system with constraints: molecular dynamics of n-alkanes. Journal of Computational Physics 1977; 23: 327-41.

Generation of stable luciferase expressing RT112 cells.

A vector containing the luciferase reporter sequence was generated by subcloning an insert BamHI/HindIII excised from pGL3 basic vector (Promega France, Charbonnières, France) into the same restriction sites of pcDNA3+ (Life Technologies). Recombinant plasmid was transfected into RT112 cells using Trans-LT1 reagent (Mirus, Madison, USA). Cells were incubated overnight, then washed twice with PBS and grown for another 24 h in RPMI + 10% SVF. Cell clones stably expressing luciferase were selected with 200 µg/ml of G418. Resistant clones were amplified and we have selected the clone showing the highest luminescence signal when incubated with luciferase substrate.

Bioluminescence in vivo imaging.

All imaging was performed under inhalational anaesthesia (3% isoflurane) and administered to a free breathing mouse using a nose cone.

For bioluminescence imaging, mice received an intraperitoneal injection of D-luciferin potassium salt dissolved in sterile phosphate-buffered serum (150 mg/kg) 5 min before imaging (ORCAII-BT-512G, Hamamatsu Photonics, Massy, France), as described previously by Jin et Al. Semi-quantitative data were obtained from the bioluminescence images by drawing regions of interest on the area to be quantified. Images were acquired as 16-bit TIFF files, which can provide a dynamic of up to 65,535 greys levels. Measurement of the bioluminescence intensities, expressed as the number of relative light units (RLU) per pixel per second for each region of interest (ROI), were performed using the Wasabi software (Hamamatsu).The colour scale values displayed by the software are adjusted to the indicated maximum values.

SUPPLEMENTARY FIGURE S1

```
Bos_taurus        MRECISIHVGQAGVQIGNACWELYCLEHGIQPDGQMPSDKTIGGGDDSFNTFFSETGAGK
Homo_sapiens      MRECISIHVGQAGVQIGNACWELYCLEHGIQPDGQMPSDKTIGGGDDSFNTFFSETGAGK
                  ************************************************************

Bos_taurus        HVPRAVFVDLEPTVIDEVRTGTYRQLFHPEQLITGKEDAANNYARGHYTIGKEIIDLVLD
Homo_sapiens      HVPRAVFVDLEPTVIDEVRTGTYRQLFHPEQLITGKEDAANNYARGHYTIGKEIIDLVLD
                  ************************************************************

Bos_taurus        RIRKLADQCTGLQGFSVFHSFGGGTGSGFTSLLMERLSVDYGKKSKLEFSIYPAPQVSTA
Homo_sapiens      RIRKLADQCTGLQGFLVFHSFGGGTGSGFTSLLMERLSVDYGKKSKLEFSIYPAPQVSTA
                  *************** ********************************************

Bos_taurus        VVEPYNSILTTHTTLEHSDCAFMVDNEAIYDICRRNLDIERPTYTNLNRLIGQIVSSITA
Homo_sapiens      VVEPYNSILTTHTTLEHSDCAFMVDNEAIYDICRRNLDIERPTYTNLNRLIGQIVSSITA
                  ************************************************************

Bos_taurus        SLRFDGALNVDLTEFQTNLVPYPRIHFPLATYAPVISAEKAYHEQLSVAEITNACFEPAN
Homo_sapiens      SLRFDGALNVDLTEFQTNLVPYPRIHFPLATYAPVISAEKAYHEQLSVAEITNACFEPAN
                  ************************************************************

Bos_taurus        QMVKCDPRHGKYMACCLLYRGDVVPKDVNAAIATIKTKRTIQFVDWCPTGFKVGINYEPP
Homo_sapiens      QMVKCDPRHGKYMACCLLYRGDVVPKDVNAAIATIKTKRTIQFVDWCPTGFKVGINYQPP
                  *********************************************************:**

Bos_taurus        TVVPGGDLAKVQRAVCMLSNTTAIAEAWARLDHKFDLMYAKRAFVHWYVGEGMEEGEFSE
Homo_sapiens      TVVPGGDLAKVQRAVCMLSNTTAIAEAWARLDHKFDLMYAKRAFVHWYVGEGMEEGEFSE
                  ************************************************************

Bos_taurus        AREDMAALEKDYEEVGVDSVEGEGEEEGEEY
Homo_sapiens      AREDMAALEKDYEEVGVDSVEGEGEEEGEEY
                  *******************************
```

Sequence alignment between α-tubulin from Bos Taurus (Q2HJ86) and from Homo sapiens (Q71U36). Sequence identity (*): 99.0%.

```
Bos_taurus          MREIVHIQAGQCGNQIGAKFWEVISDEHGIDPTGSYHGDSDLQLERINVYYNEAAGNKYV
Homo_sapiens        MREIVHIQAGQCGNQIGAKFWEVISDEHGIDPTGSYHGDSDLQLERINVYYNEATGNKYV
                    ******************************************************:*****

Bos_taurus          PRAILVDLEPGTMDSVRSGPFGQIFRPDNFVFGQSGAGNNWAKGHYTEGAELVDSVLDVV
Homo_sapiens        PRAILVDLEPGTMDSVRSGPFGQIFRPDNFVFGQSGAGNNWAKGHYTEGAELVDSVLDVV
                    ************************************************************

Bos_taurus          RKESESCDCLQGFQLTHSLGGGTGSGMGTLLISKIREEYPDRIMNTFSVVPSPKVSDTVV
Homo_sapiens        RKESESCDCLQGFQLTHSLGGGTGSGMGTLLISKIREEYPDRIMNTFSVMPSPKVSDTVV
                    *************************************************:**********

Bos_taurus          EPYNATLSVHQLVENTDETYCIDNEALYDICFRTLKLTTPTYGDLNHLVSATMSGVTTCL
Homo_sapiens        EPYNATLSVHQLVENTDETYCIDNEALYDICFRTLKLTTPTYGDLNHLVSATMSGVTTCL
                    ************************************************************

Bos_taurus          RFPGQLNADLRKLAVNMVPFPRLHFFMPGFAPLTSRGSQQYRALTVPELTQQMFDAKNMM
Homo_sapiens        RFPGQLNADLRKLAVNMVPFPRLHFFMPGFAPLTSRGSQQYRALTVPELTQQMFDSKNMM
                    *******************************************************:****

Bos_taurus          AACDPRHGRYLTVAAVFRGRMSMKEVDEQMLNVQNKNSSYFVEWIPNNVKTAVCDIPPRG
Homo_sapiens        AACDPRHGRYLTVAAIFRGRMSMKEVDEQMLNVQNKNSSYFVEWIPNNVKTAVCDIPPRG
                    ***************:********************************************

Bos_taurus          LKMSATFIGNSTAIQELFKRISEQFTAMFRRKAFLHWYTGEGMDEMEFTEAESNMNDLVS
Homo_sapiens        LKMSATFIGNSTAIQELFKRISEQFTAMFRRKAFLHWYTGEGMDEMEFTEAESNMNDLVS
                    ************************************************************

Bos_taurus          EYQQYQDATADEQGEFEEEGEEDEA
Homo_sapiens        EYQQYQDATADEQGEFEEEEGEDEA
                    *******************  ****
```

Sequence alignment between β-tubulin from Bos Taurus (Q2HJ81) and from Homo sapiens (Q9BVA1). Sequence identity (*): 99.0%.

SUPPLEMENTARY FIGURE S2

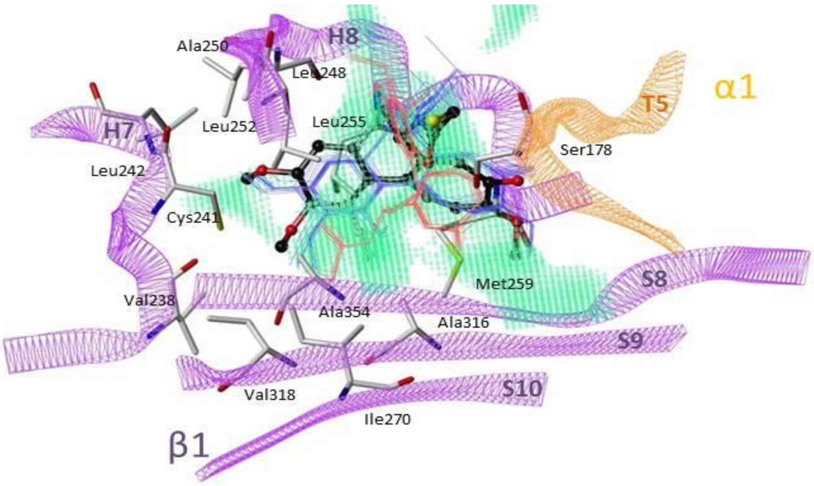

A

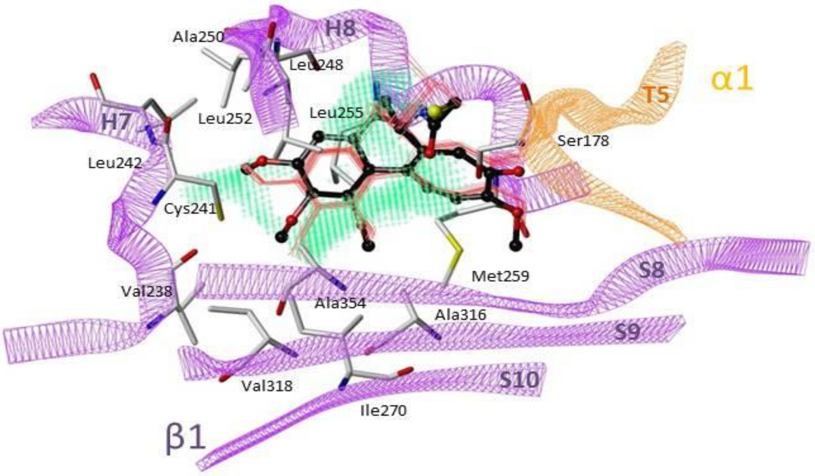

B

Re-docking of DAMA colchicine (represented in black as ball and sticks) in the colchicine binding domain of tubulin.

β-subunit is represented as purple ribbons whereas the α-subunit of the same heterodimer is represented as orange ribbons. Key residues are represented as sticks and labeled in black. **A**, Docking results obtained with the standard GOLD docking approach. Hydrophobic regions of the pocket, defined *by default*, are represented as green dots. The docking best-ranked poses are represented as red lines whereas the docking poses belonging to the most populated cluster are in blue. **B**, Docking results obtained with the MLP-filter docking approach for which a clear convergence of docking poses towards the crystallographic one was obtained. Hydrophobic regions of the pocket, defined according to the MLP, are represented as green dots. The docking best-ranked poses are represented as red lines.

LEGENDS OF SUPPLEMENTARY MOVIE S1 AND MOVIE S2

Supplementary movie S1: Time lapse imagery on mitotic GFP-tubulin expressing HeLa cells in control medium without IPP51.

Supplementary movie S2: Time lapse imagery on mitotic GFP-tubulin expressing HeLa cells exposed to 10 µM IPP51.
